# Nitric oxide–centred signaling networks in plants: from growth to postharvest

**DOI:** 10.3389/fpls.2026.1924602

**Published:** 2026-08-20

**Authors:** Michaela Sedlářová, Tereza Jedelská, Aleš Lebeda, Marek Petřivalský

**Affiliations:** 1 Department of Botany, Faculty of Science, Palacký University in Olomouc, Olomouc, Czechia; 2 Department of Biochemistry, Faculty of Science, Palacký University in Olomouc, Olomouc, Czechia

**Keywords:** fruit senescence, NO signaling, phytohormones, redox homeostasis, shelf life

## Abstract

Nitric oxide (NO) has emerged as a versatile signaling molecule that regulates plant development, stress responses, and redox homeostasis through interactions with reactive oxygen species (ROS), phytohormones, and other bioactive compounds. In horticulture, postharvest losses remain a major challenge due to ongoing metabolic activity associated with ripening and senescence. Increasing evidence identifies NO as a key regulator capable of mitigating these processes and extending shelf life. This Mini Review provides a concept-driven synthesis positioning NO as a central integrator of plant signaling networks. We highlight its role as a redox–hormonal hub coordinating interactions with ROS and phytohormones. Special emphasis is placed on the dual role of NO as both a signaling molecule and a modulator of nitro-oxidative stress during plant growth, fruit ripening, and postharvest physiology. We further discuss key molecular mechanisms underlying postharvest responses, together with current controversies related to NO biosynthesis, concentration-dependent effects, and practical application strategies.

## 1 Introduction

Post-harvest quality of plant-derived products is influenced by ongoing metabolic activity, including respiration, loss of water, hormone-driven maturation, senescence, and biotic as well as abiotic stressors (Khator et al., 2024; Tipu and Sherif, 2024). These processes are closely associated with imbalances in redox homeostasis and hormonal regulation, ultimately leading to food quality deterioration and reduced shelf life. In this context, attention has been directed towards small gasotransmitters (Corpas et al., 2025a; Wu et al., 2025) and particularly nitric oxide (NO) and ethylene (ET) as regulators capable of modulating postharvest physiology and mitigating economic losses (Sun et al., 2021; Muñoz-Vargas et al., 2023; Zhong et al., 2024).

**Figure 1:**
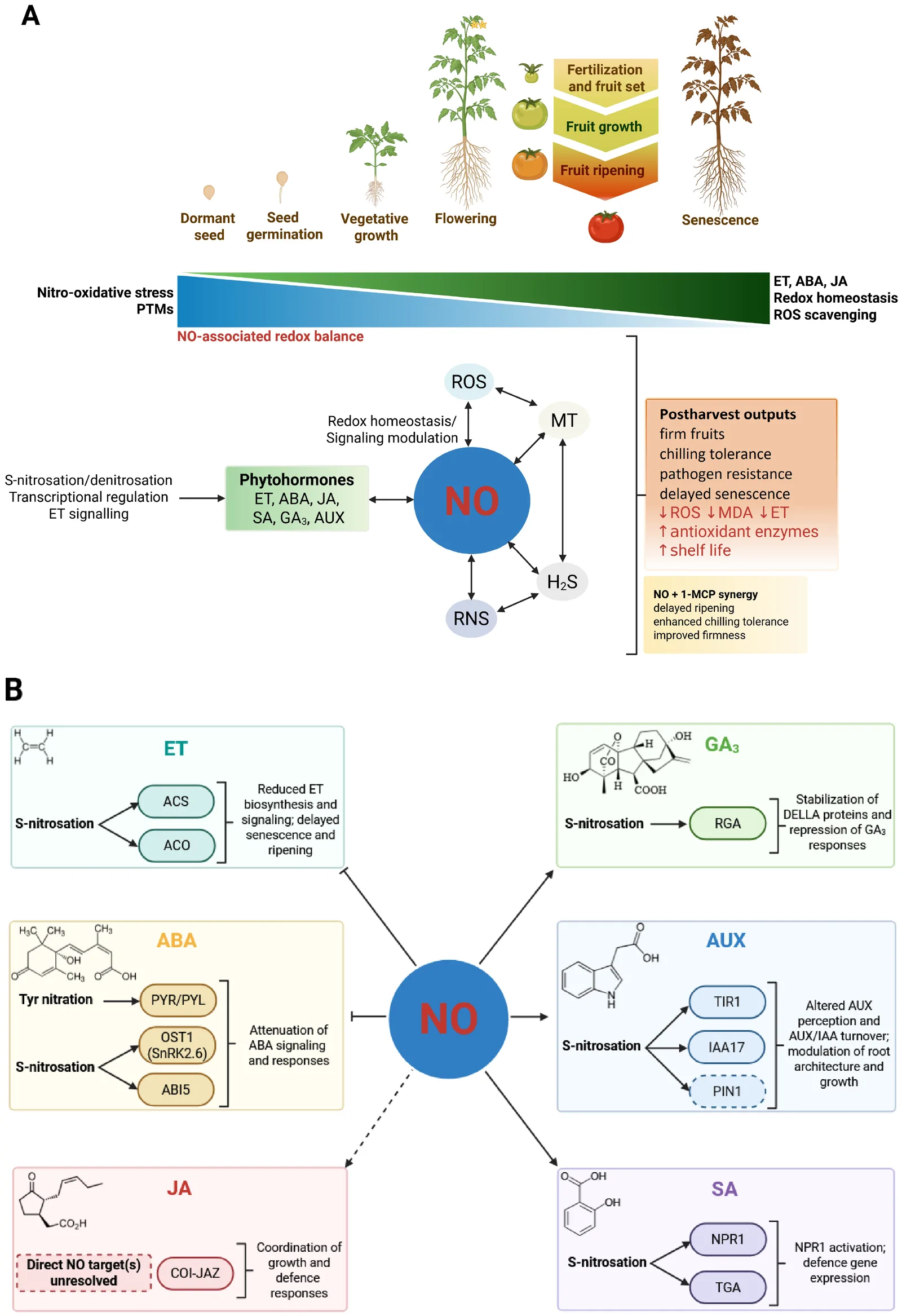
**(A)** Nitric oxide (NO) as a central redox–hormonal integrator coordinating plant development, ripening, and postharvest physiology. The schematic overview illustrates the multifaceted role of NO across major developmental stages of horticultural crops, from seed dormancy and germination through vegetative growth, flowering, fruit development, ripening, and senescence. NO operates as a central signaling hub integrating reactive oxygen species (ROS), reactive nitrogen species (RNS), hydrogen sulphide (H_2_S), melatonin (MT), and phytohormone-dependent pathways, including ethylene (ET), abscisic acid (ABA), jasmonic acid (JA), salicylic acid (SA), gibberellic acid (GA_3_), and auxin (AUX). Through redox-dependent signaling, post-translational modifications (PTMs), transcriptional regulation, and modulation of ET signaling, NO contributes to the maintenance of redox homeostasis and antioxidant capacity while limiting nitro-oxidative stress. In postharvest systems, NO-mediated signaling is associated with improved fruit firmness, chilling tolerance, enhanced pathogen resistance, delayed senescence, reduced ROS, malondialdehyde (MDA), and ET, as well as increased antioxidant enzymes activities and extended shelf life. The figure also highlights the synergistic interaction between NO and 1-methylcyclopropene (1-MCP), which contributes to delayed ripening, improved firmness, and enhanced chilling tolerance during storage. **(B)** Representative molecular mechanisms of NO – phytohormone crosstalk. NO regulates key signaling components of ET, ABA, JA, GA_3_, AUX, and SA pathways through tyrosine nitration and S-nitrosation, thereby modulating hormone perception, signaling, protein stability, and downstream physiological responses. Solid and dashed lines represent experimentally supported and proposed interactions, respectively. NO, nitric oxide; ABA, abscisic acid; AUX, auxin; GA_3_, gibberellic acid; SA, salicylic acid; JA, jasmonic acid; ET, ethylene; ACS, 1-aminocyclopropane-1-carboxylic acid synthase; ACO, 1-aminocyclopropane-1-carboxylic acid oxidase; ABI5, ABA-insensitive 5; OST1, OPEN STOMATA 1 (SnRK2.6); PYR/PYL, PYRABACTIN RESISTANCE/PYR1-LIKE ABA receptors; TIR1, TRANSPORT INHIBITOR RESPONSE 1; IAA17, AUX/IAA protein 17; PIN1, PIN-FORMED 1 auxin efflux carrier; RGA, REPRESSOR OF ga1-3 (DELLA protein); NPR1, NONEXPRESSOR OF PATHOGENESIS-RELATED GENES 1; TGA, TGACG motif-binding transcription factor; COI1, CORONATINE INSENSITIVE 1; JAZ, JASMONATE ZIM-DOMAIN protein. Created in https://BioRender.com.

Numerous studies report NO key role in plant signaling throughout seed germination (Bykova and Igamberdiev, 2025; Bakhshy et al., 2026), development (Khan et al., 2026), stress responses (He et al., 2023; Kumari et al., 2024; Naaz et al., 2025; Dev et al., 2026), fruit ripening and senescence (Kolbert et al., 2021a), including its interactions with reactive oxygen species (ROS) (Jedelská et al., 2021a; Wani et al., 2021), hydrogen (Hancock et al., 2026), hydrogen sulphide (H_2_S) (Corpas, 2024), melatonin (MT) and phytohormones (**Figure 1**). Herein, NO is discussed as a central integrator of signaling networks across plant ontogeny, with insights into the underlying molecular mechanisms, e.g. transcriptional regulation and posttranslational modifications (PTMs). By integrating current knowledge into a unified conceptual framework, this mini-review aims to bridge fundamental plant signaling with applied horticultural research. We further highlight key challenges and future directions toward the development of NO-based strategies for promoting sustainable postharvest management.

## 2 NO biosynthesis as a flexible and context-dependent network

NO, as a small reactive gaseous molecule, was proposed to be emitted by plants in 1960. After the pioneering studies on NO emission from plants proven by Lowell Klepper (Klepper, 1979), NO arose as a pivotal regulator of plant physiology in 1990´s (Hancock et al., 2025; Kolbert et al., 2026). NO production has been documented so far in plant peroxisomes, cytoplasm, mitochondria, chloroplasts, plasma membrane and apoplast (reviewed in León and Costa-Broseta, 2020; Sandalio et al., 2021), though its precise origin remains a matter of ongoing debate. Comprehensive overviews of NO production and metabolism have been published elsewhere (Liu and Liao, 2025; Sedlářová et al., 2025). Its most widely recognised plant enzymatic source, nitrate reductase (NR), can produce NO from nitrite under high cytosolic nitrite concentration and low-oxygen levels (Mohn et al., 2019; Gupta et al., 2025). Despite that, similarly to animals, L-arginine-dependent nitric oxide synthase (NOS)-like activities have been reported in plants, the absence of a clearly identified canonical NOS enzyme in higher plants remains the major unresolved issue (Corpas et al., 2022). Alternative pathways described to contribute to NO production include mitochondrial electron transport chain-linked NO reductase, polyamine metabolism, hydroxylamine oxidation, oxime conversion, and non-enzymatic nitrite reduction under low pH (Allagulova et al., 2023; López-Gómez et al., 2024; Corpas et al., 2026).

Maintaining NO homeostasis is essential due to its high reactivity and dual role in signaling and stress responses (Sedlářová et al., 2025). S-nitrosoglutathione (GSNO) represents the major reservoir and transport form of NO. The enzyme S-nitrosoglutathione reductase (GSNOR) regulates GSNO turnover and intracellular levels, and thereby controls the extent of protein S-nitrosation and overall nitrosative signaling (Das et al., 2025a; Jedelská et al., 2026). Through this mechanism, NO bioavailability is tightly interconnected with cellular redox status, glutathione metabolism (Jedelská et al., 2019; Treffon et al., 2021), and hormone homeostasis (Jing et al., 2023; Zuccarelli et al., 2023; Liu and Liao, 2025).

## 3 NO as an integrator of plant signaling networks

NO is increasingly recognised not merely as an individual signaling molecule, but as a central coordinator of plant signaling networks that integrates redox status with hormonal and metabolic regulation (Kolbert et al., 2024, Kolbert et al., 2026).

NO and ROS form a dynamic redox network in which these reactive molecules can act synergistically or antagonistically depending on their relative concentrations, subcellular localization, and environmental conditions (Romero-Puertas et al., 2021; Khan et al., 2023; Kolupaev et al., 2023; Gul et al., 2026; Hancock et al., 2026). NO influences ROS homeostasis through antioxidant defence systems, including superoxide dismutase (SOD), catalase (CAT), and enzymes of the ascorbate–glutathione cycle, thereby maintaining cellular redox equilibrium partly through covalent PTMs such as cysteine S-nitrosation and tyrosine nitration (Palma et al., 2020; Jedelská et al., 2021b; Borrowman et al., 2023; Corpas et al., 2025b). PTMs can suppress ROS production and enhance antioxidant capacity (Zhang et al., 2025b). Conversely, excessive accumulation of NO and ROS leads to nitro-oxidative stress, promoting cellular damage and accelerating senescence (Kolbert et al., 2021a; Sandalio et al., 2023). In addition, NO has been proposed to regulate aquaporins (peroxiporins) involved in the intercompartmental movement of hydrogen peroxide (H_2_O_2_), as well as mitogen-activated protein kinase (MAPK) cascades associated with stress signaling and acclimation (Mata-Pérez et al., 2023; Mukherjee et al., 2024; Jia et al., 2025). Through coordinated effects on transcription factors (TFs), MAPK signaling, aquaporins, and hormone-dependent pathways, NO–ROS interactions can exert both positive or negative effects on plant development, stress acclimation, and defence responses (Khedr and Alomran, 2026).

Integrative function of NO is evident from its extensive crosstalk with a wide range of molecules beyond ROS e.g., H_2_S, MT, phytohormones, particularly during ontogenetic transitions such as fruit ripening and senescence (**Figures 1A, B**), where redox status and hormonal signaling must be tightly coordinated (Li et al., 2022). In the following text, we focus on ethylene (ET), abscisic acid (ABA), jasmonic acid (JA), gibberellic acid (GA_3_), auxin (AUX), salicylic acid (SA), and melatonin (MT), which themselves are closely linked to ROS signaling and metabolism (Devireddy et al., 2021). Linkage of NO to other molecules essential for plant (patho)physiology is also coming to the forefront of interest. For instance, NO-activated GABA shunt and antioxidant systems enhance peach resistance to fruit rot during peach storage (Pan et al., 2023). Crosstalk between NO and H_2_S is mediated through PTMs, such as S-nitrosation and persulfidation, which target overlapping sets of proteins and modulate their activity in a context-dependent manner (Mishra et al., 2021; Muñoz-Vargas et al., 2023; Corpas et al., 2026; Mohanty et al., 2026).

## 4 Plant NO in postharvest physiology

High diffusivity and reactivity enable NO to coordinate cellular homeostasis, division, organ differentiation and developmental transitions (Kolbert et al., 2024; da Silva et al., 2024; Lei et al., 2025; Sarma et al., 2025). This regulatory role extends beyond active plant growth; NO interacts with phytohormones during fruit ripening, delays postharvest senescence, reduces chilling damage and enhances resistance to diseases of stored crops (Ren et al., 2020; Wang et al., 2020; Zhong et al., 2024);. Thus, NO can be utilised to regulate stomatal closure, photosynthesis and defence mechanisms (Corpas et al., 2025b; Kuźniak and Ciereszko, 2025). NO-dependent ROS accumulation reduces lipid peroxidation, preserves membrane and cell wall integrity, thus delaying senescence and extending commodity shelf life (Gouda et al., 2025; Siddiqui et al., 2024).

### 4.1 NO–ethylene interactions

ET, a central regulator of climacteric fruit ripening, orchestrates respiration, cell wall disassembly, pigment accumulation, and senescence (Tipu and Sherif, 2024). NO modulates both ET biosynthesis and signaling, thereby delaying ripening and improving postharvest performance (Sougrakpam et al., 2023; Maurya et al., 2025; Wei et al., 2025; Khedr and Alomran, 2026; Thewes et al., 2026).

At the biosynthetic level, NO suppresses ET production primarily through modulation of 1-aminocyclopropane-1-carboxylate synthase (ACC synthase, ACS) and 1-aminocyclopropane-1-carboxylate oxygenase (ACC oxidase, ACO), the key enzymes responsible for ET formation (Wei et al., 2022). Both transcriptional regulation and PTMs, particularly S-nitrosation, contribute to the inhibition of ACS and ACO activity (Li et al., 2025). In peach and papaya, S-nitrosation of ACO proteins reduced ET emission, while in tomato, S-nitrosation of methionine adenosyltransferase (MAT) limited substrate availability for ET biosynthesis (Li et al., 2022; Liu et al., 2023a). In addition, NO modulates ethylene-responsive TFs (ERFs) (Ji et al., 2022; Wu et al., 2025) in a manner similar to GA_3_ (Lin et al., 2025). In apple, NO-activated MdERF5 repressed ET biosynthetic genes and delayed ripening (Ji et al., 2022).

Increasing attention focused on interactions between NO and commercial ET inhibitor 1-methylcyclopropene (1-MCP), used to delay ripening and thus extend the shelf life of climacteric fruits (Wu et al., 2021). While 1-MCP blocks ET perception at the receptor level, NO exerts broader effects by modulating ET biosynthesis, redox homeostasis, and antioxidant metabolism simultaneously (Qiao et al., 2025). Combined treatments with NO donors and 1-MCP can produce synergistic effects, e.g. GSNO enhanced the beneficial effects of 1-MCP in blueberry by improving firmness and ascorbic acid retention during cold storage (Gergoff Grozeff et al., 2017), whereas combined NO donor and 1-MCP treatments extended tomato shelf-life through suppression of ET biosynthesis and activation of antioxidant enzymes (Steelheart et al., 2019).

Importantly, NO–ET crosstalk is tightly interconnected with ROS metabolism (Corpas, 2024). The climacteric surge in ET production is typically accompanied by increased ROS generation, contributing to oxidative stress and senescence (Wang et al., 2025). At moderate concentrations, NO enhances antioxidant defence systems, including the activities of SOD, CAT, and the enzymes of the ascorbate–glutathione cycle (Liu et al., 2021). S-nitrosation and tyrosine nitration further provide a mechanistic link between NO, ROS, and ET signaling, fine-tuning the balance between ripening progression and tissue deterioration. However, excessive NO or ROS accumulation may shift this balance towards nitro-oxidative stress and quality loss (Corpas et al., 2025b). The NO–ET–ROS module represents the major central regulatory node during postharvest storage (Qiao et al., 2025; Azzawi et al., 2026).

### 4.2 NO–abscisic acid crosstalk

NO exhibits a complex interaction with ABA, a hormone central to stress responses and senescence (Zhai et al., 2026). During seed germination, NO promotes ABA catabolism, thereby reducing ABA levels and facilitating the release of seed dormancy (Šírová et al., 2011). At the molecular level, NO negatively regulates ABA signalling by promoting S-nitrosation and degradation of key positive regulators, including SnRK2 protein kinases and the transcription factor ABI5, and nitration of PYR/PYL ABA receptors (León and Costa-Broseta, 2020). In postharvest systems, ABA–NO crosstalk plays an important role, particularly under cold storage conditions. In peach, ABA stimulates sucrose metabolism (Zhao et al., 2026), while ABA-induced NO production via the NR pathway enhances antioxidant defence systems and alleviates chilling disorders (Tian et al., 2020). Beyond cold stress acclimation, ABA–NO interactions also contribute to ripening through redox-dependent regulation of ET biosynthesis. While ABA generally promotes ET biosynthesis, NO can counteract this effect via S-nitrosation of ACO (Qi et al., 2025). In addition, ABA-induced NO signalling contributes to stomatal closure through modulation of ion channel activity and guard cell turgor (Li et al., 2026), thereby maintaining tissue hydration during storage.

### 4.3 NO–jasmonate crosstalk

Jasmonic acid (JA) and methyl jasmonate (MeJA) signaling is vital for plant defence to injury and abiotic stress. JA and MeJA together with NO operate through interconnected signaling pathways coordinating redox homeostasis and ET-associated senescence processes (Li et al., 2023; Mu et al., 2025). JA signaling is coordinated by the COI1–JAZ module, while recent evidence further identifies JAZ1 as an integrative hub linking JA signaling with other regulatory pathways involved in balancing growth and defence (Pu et al., 2025).

MeJA treatments delayed softening and suppressed ET production in kiwifruit (Allegra et al., 2025), and enhanced antioxidant capacity during papaya storage (Li et al., 2023). Direct evidence for NO–JA synergy was demonstrated in tomato fruit, where combined MeJA and NO donor (SNP) application reduced chilling disorders, maintained firmness, suppressed respiration and ET production, and elevated antioxidant capacity more compared with individual treatments (Padilla-González et al., 2025).

Direct evidence for NO–JA/MeJA interactions in postharvest systems remains limited. Nevertheless, a study in blueberry fruit demonstrated that NO accumulation is required for MeJA-induced resistance against *Botrytis cinerea*, whereas NO scavenging largely abolished ROS signaling and disease resistance responses (Wang et al., 2020).

### 4.4 NO–gibberellin interactions in postharvest fruit quality maintenance

Increasing evidence suggests that NO modulates GA_3_ signaling in a context-dependent manner during, e.g. dormancy, seed germination, stem elongation, photosynthesis, fruit development, and stress adaptation through integration with redox-dependent pathways (Azzawi et al., 2026). NO influences GA_3_ biosynthesis, especially under extreme conditions such as aluminium toxicity (Lu et al., 2024; Jiang et al., 2026) and salt stress (Chen et al., 2022). NO negatively regulates GA_3_ signaling through S-nitrosation of the DELLA protein RGA at Cys374 in *A. thaliana*, preventing its proteasomal degradation, and suppressing GA_3_ responses (Chen et al., 2022).

Applied via pre-harvest sprays or postharvest dips, GA_3_ stimulates antioxidant capacity, phenolic metabolism, and delays fruit senescence by manipulating ET biosynthesis as shown e.g. in peach (Sun et al., 2024) and apples (Lin et al., 2025). Recent transcriptomic analyses of grapes further revealed regulation of TFs and genes, indicating that NO and GA_3_ signaling converge on the regulation of cell wall strengthening and ROS homeostasis. Their preharvest application contributes to fruit firmness and delayed postharvest senescence (Hu et al., 2024). Combined application of GA_3_ and NO donor also prolongs the vase-life of flowers (see e.g., Chauhan et al., 2024).

### 4.5 NO–auxin crosstalk in fruit ripening

NO is closely interconnected with AUX signaling during plant development and stress responses (Singh et al., 2026). It is known to function as a downstream mediator regulating root system architecture (Das et al., 2025b) as well as fruit set (Zuccarelli et al., 2021). NO influences AUX levels through both transcriptional regulation and PTMs (Jing et al., 2023; Cui et al., 2024; Jedelská et al., 2026), affecting AUX biosynthesis, degradation, conjugation, allocation, signalling, and AUX influx (reviewed in Sarma et al., 2025). Mechanistically, NO regulates AUX signaling through S-nitrosation of TIR1 receptor and transcriptional repressor IAA17, thereby modulating AUX perception, protein turnover, and downstream AUX responses (Jing et al., 2023).

The multifaceted process of fruit ripening is orchestrated by AUX in various ways. In climacteric fruits, AUX inhibits the ripening of tomatoes whereas it stimulates the ripening of apples and peaches (Zhang et al., 2025a). AUX signaling components such as *AUX/IAA* and *SAUR* genes are differentially regulated during storage, e.g. PpSAUR43 acts as a negative regulator of the peach post-ripening process (Wang et al., 2022). AUX influences the ripening of non-climacteric fruits (citruses, grapes, strawberries) independently of ET through expression of genes linked with colour changes and sugar accumulation (Zhang et al., 2025a). Although increasing evidence suggests that NO–AUX molecular crosstalk exists during ripening and postharvest physiology, these remain to be elucidated.

### 4.6 NO–salicylic acid crosstalk in defence and postharvest regulation

SA, in postharvest physiology mainly applied as methyl salicylate (MeSA), is a key regulator of plant defence under biotic stress (Tian et al., 2025). SA and NO co-treatments maintain fruit postharvest quality, though application of solely SA in cherries demonstrated superior storage performance (Zhang et al., 2023). Current evidence supports the view that SA–NO crosstalk coordinates activation of antioxidant systems, enhances stress tolerance, and delays postharvest senescence (Li et al., 2022; Liu et al., 2023b; Cheng et al., 2025). NO regulates SA signaling through reversible S-nitrosation of the master immune regulator NPR1, thereby controlling its interaction with TGA transcription factors and activation of defence-related genes (Borrowman et al., 2023). However, the physiological outcomes of SA–NO interactions remain strongly dependent on crop species, developmental stage, and application conditions, highlighting the need for further mechanistic and application-oriented studies.

### 4.7 NO–MT crosstalk

Growing evidence indicates that MT, a phytohormone-like regulator of metabolism, interacts with NO both as a direct antioxidant and as a regulator of NO biosynthesis and signaling, contributing to the fine-tuning of photosynthesis, stress responses and senescence (Xu et al., 2023; Cai et al., 2024; Liu and Liao, 2025; Taboada et al., 2026; Witoszek et al., 2026). Recent findings support a model in which MT upregulates NR expression, while NO executes various signaling functions together with stimulation of MT through the cGMP signaling pathway, which creates a mutually reinforcing feedback loop (Lee et al., 2025). In postharvest systems, N-nitrosomelatonin might be utilized as a prospective donor of NO and MT to delay senescence through interactions with AUX, ABA and SA (Zhu et al., 2019; Martínez-Lorente et al., 2022; Hussain et al., 2024).

## 5 Knowledge gaps, controversies, and challenges

Advances in knowledge of postharvest physiology and technology enables to prevent deterioration of horticultural and agricultural products by senescence and biotic and abiotic diseases (Qiao et al., 2025). Starting four decades ago with studies aiming to unveil individual components of NO pathways and the effect of solely NO external application on fruits there is a trend to examine plant metabolism in whole complexity and combine NO applications with other molecules, namely phytohormones (**Table 1**). Despite substantial progress in understanding NO signaling in plants, several fundamental questions and practical limitations remain unresolved:

**Table 1:** Overview of key studies reporting combined external application of NO and phytohormones and their effects on postharvest quality.

Phytohormone	NO donor	Plant species	Positive effects	Reference
ET inhibitor 1-MCP	GSNO	*Vaccinium corymbosum*	Delayed senescence, maintained firmness, enhanced antioxidant capacity (ascorbic acid)	Gergoff Grozeff et al. (2017)
		*Solanum lycopersicum*	Enhanced antioxidant capacity (ROS metabolism enzymes)	Steelheart et al. (2019)
GA_3_	SNP	*Vitis vinifera*	Delayed senescence, increased firmness (complex interactions in cell wall metabolism, ROS homeostasis)	Hu et al. (2024)
		*Dahlia variabilis*	Enhanced antioxidant capacity (ROS metabolism enzymes, higher anthocyanin content)	Chauhan et al. (2024)
SA	SNP	*Prunus avium*	Enhanced antioxidant capacity (ROS metabolism enzymes, higher phenolic content), resistance to fungal pathogens	Zhang et al. (2023)
MeSA	SNP	*Solanum lycopersicum*	Reduced chilling injury, maintained firmness, enhanced antioxidant capacity (ROS metabolism enzyme APX, higher phenolic content)	Padilla-González et al. (2025)
ABA	NO	*Prunus persica*	Reduced chilling injury, enhanced antioxidant capacity, improved cold tolerance	Tian et al. (2020)
	SNP	*Rosa hybrida*	Delayed senescence, increased flower diameter, reduced water loss; enhanced antioxidant capacity (ROS metabolism enzymes)	Deng et al. (2019)
MeJA	SNP	*Solanum lycopersicum*	Reduced chilling injury, maintained firmness, enhanced antioxidant capacity (ROS metabolism enzyme APX, higher phenolic content)	Padilla-González et al. (2025)
		*Cucumis sativus*	Reduced chilling injury, enhanced antioxidant capacity (ROS metabolism enzyme CAT)	Liu et al. (2016)
MT	SNP	*Pyrus communis*	Delayed senescence, maintained firmness, reduced ET production	Liu et al. (2019)

ABA, abscisic acid; APX, ascorbate peroxidase; CAT, catalase; ET, ethylene; GA_3_, gibberellic acid; GSNO, S-nitrosoglutathione; MeJA, methyl jasmonate; MeSA, methyl salicylate; MT, melatonin; NO, nitric oxide; ROS, reactive oxygen species; SNP, sodium nitroprusside; 1-MCP, 1-methylcyclopropene.

1. NO biosynthesis and regulation, e.g., relative contribution of individual production pathways, their cellular compartmentalisation, and subtle regulation in individual plant species under varying developmental and environmental conditions;
2. Disentangling crosstalk with ROS, H_2_S, and phytohormones at the molecular level, with overlapping targets of PTMs such as S-nitrosation and persulfidation;
3. Accurate, specific, and sensitive detection and quantification methods of NO and related RNS, as commonly used methods are prone to artifacts;
4. Integration of multi-omics approaches into NO research;
5. Delivery of NO as a ripening suppressor: NO gas fumigation, NO-donors SNP, GSNO, and chitosan encapsulated nanoparticles differ in release kinetics, stability, and potential side effects (toxic by-products and residue formation);
6. A gap between controlled experimental conditions and horticultural growing systems.

## 6 Future perspectives

Conceptualising NO as a dynamic redox–hormonal switch controlling the transition between growth, ripening, senescence, and stress responses opens new opportunities to improve crop performance and support sustainable postharvest management. Targeting key components of NO metabolism and signaling through breeding, biotechnology (Wulf et al., 2026), or optimised postharvest treatments (Kolbert et al., 2021b; Seabra et al., 2022; Wu et al., 2025; Thewes et al., 2026) may enhance stress resilience and extend shelf life in horticultural crops. Innovations in the distribution of plant products, such as smart packaging based on nanomaterial indicator labels that change colours in the presence of marker gas (ET, CO_2_) enable tracking freshness over time easily (Du et al., 2025). Modified atmosphere packaging already studied with ET inhibitor 1-MCP (Hou et al., 2026) might be advanced with optimized NO delivery systems, including NO gas, controlled-release NO donors, nanomaterial-based carriers, or co-treatments with other molecules (N-nitrosomelatonin). Tailoring these strategies to individual crops, developmental stages, and storage conditions will be critical for maximising beneficial effects while minimising risks associated with nitro-oxidative stress.
